# Supplementary material for: Effective EMI shielding behaviour of thin graphene/PMMA nanolaminates in the THz range
Source: Nat Commun. 2021 Aug 2;12:4655. doi: 10.1038/s41467-021-24970-4 (PMC8329220; doi:10.1038/s41467-021-24970-4)
Supplement: Supplementary file 1 — Supplementary Information [file 41467_2021_24970_MOESM1_ESM.pdf]

Supplementary Information

Effective EMI shielding behaviour of thin graphene/ PMMA nano-laminates in the THz range

*Christos Pavlou, Maria Giovanna Pastore Carbone, Anastasios C. Manikas, George Trakakis, Can Koral, Gianpaolo Papari, Antonello Andreone and Costas Galiotis**

*Corresponding author: c.galiotis@iceht.forth.gr, galiotis@chemeng.upatras.gr

This PDF file includes:

- Supplementary Discussion
- Supplementary Methods
- Supplementary Figures 1 to 7
- Supplementary Tables 1 to 3
- Supplementary References

Supplementary Discussion

Mechanical model of CVD graphene/polymer nanolaminates and derivation of the effective modulus of graphene

In the micromechanics approach, the main proposed strategies to predict the effective properties of a composite material are based on the implementation of analytical methods (such as the Rule of Mixtures [1]) or semi-empirical methods (such as the Halpin-Tsai model [2]).

One of the simplest relationships that has been developed to describe the reinforcement achieved from a high-modulus filler in a low-modulus matrix, under uniform strain, is the so-called "rule of mixtures" (RoM), in which the Young's modulus of a composite E_c along the fibres direction is given by

$$E_{\mathrm{c}} = E_{\mathrm{m}}(1 - V_{\mathrm{f}}) + V_{\mathrm{f}}E_{\mathrm{f}} \qquad \text{Supplementary Equation (1)}$$

where E_{m} and E_{f} are respectively the modulus of the matrix and of the filler, and V_{m} and V_{f} are the volume fraction of the matrix and of the filler. The RoM was developed for continuous and unidirectional fibres and its simplicity is coming from hypothetical assumptions such as the unidirectional alignment of the fibres, their infinite length and the perfect bonding between the components.

Another common approach used for nanocomposites with parallel-aligned nanoplatelets is the Halpin-Tsai model (HT). Accordingly, for nanocomposites having fillers with high orientation degree, the Young's modulus in the direction longitudinal to the filler orientation (E_{II}) can be estimated by:

$$E_{\mathrm{II}} = \left[\frac{1+2a\eta_{\mathrm{II}}V_{\mathrm{f}}}{1-\eta_{\mathrm{II}}V_{\mathrm{f}}}\right]E_{\mathrm{m}} \qquad \text{Supplementary Equation (2)}$$

where, η_{II} is defined as

$$\eta_{\mathrm{II}} = \left[\frac{\frac{E_{\mathrm{f}}}{E_{\mathrm{m}}}-1}{\frac{E_{\mathrm{f}}}{E_{\mathrm{m}}}+2a}\right] \qquad \text{Supplementary Equation (3)}$$

and a is the aspect ratio of the filler (width/thickness).

It is interesting noting that, when $a \to \infty$, which is the case of very large filler platelets, Supplementary Equation (2) is then reduced to the RoM and gives the maximum reinforcement:

$$E_{\mathrm{II}} = E_{\mathrm{f}} V_{\mathrm{f}} + E_{\mathrm{m}}(1 - V_{\mathrm{f}}) \qquad \text{Supplementary Equation (2-i)}$$

We implemented the HT model to predict the Young's modulus of a PMMA-graphene composite with fully aligned layers, with different aspect ratios, a. Results are plotted in Supplementary Figure 2 as a function of graphene volume fraction and are compared to the prediction based on the RoM. In the models, E_{f} and E_{m} have been set, respectively, as 1.01 TPa and 1.75 GPa. The former is modulus of pristine, defect-free graphene [3] and the latter is the modulus of PMMA as evaluated on the bases of our tensile tests. It is clear that the reinforcement approaches the maximum reinforcement when a >10,000, which is the point at which the HT model approaches the RoM, in agreement with the analysis reported by Liu et al. [4].

The aspect ratio of the proposed nanolaminates is higher than 1,000,000; therefore the RoM can be safely adopted to validate our results. Actually, in order to derive the effective modulus of graphene in the nanolaminate system, the RoM can be rewritten as follows:

$$E_{\mathrm{c}} = E_{\mathrm{m}}(1 - V_{\mathrm{f}}) + V_{\mathrm{f}} E_{\mathrm{f}} \quad \Rightarrow \quad E_{\mathrm{c}} = E_{\mathrm{m}} - E_{\mathrm{m}} V_{\mathrm{f}} + V_{\mathrm{f}} E_{\mathrm{f}}$$

$$\Rightarrow \quad E_{\mathrm{c}} = E_{\mathrm{m}} + V_{\mathrm{f}}(E_{\mathrm{f}} - E_{\mathrm{m}}) \qquad \text{Supplementary Equation (1-i)}$$

Therefore, E_{f} can be derived by applying a linear square fitting to the experimental data E_{c} plotted as a function of V_{f}. In fact, Supplementary Equation (1-i) is in the form $y = a + bx$ and the effective modulus of graphene E_f is obtained from the slope term:

$$b = (E_{\mathrm{f}} - E_{\mathrm{m}}) \quad \Rightarrow \quad E_f = E_{\mathrm{m}} + b$$

Theoretical Calculation of EMI Shielding Effectiveness

a) Minimum shielding performance

For electrically thin samples with $t << \delta$ (skin depth), and under the good conductor approximation, the total shielding effectiveness can be expressed as [5]:

$$\mathrm{SE_T} = 20\log_{10}\left(1 + \frac{Z_0}{2}\sigma t\right) \qquad \text{Supplementary Equation (4)}$$

where Z_0 is the free-space impedance and σt is the inverse of the sample sheet resistance (σ is the DC conductivity).

This formula can be easily extended in the case of graphene/polymer multilayers introducing the sheet number N (>1) instead of the sample thickness as parameter [6].

Values extracted using this simple model allows to predict the "zero-frequency" shielding performance of our samples. The numerical results have been reported in Supplementary Table 3 and nicely match but for the smallest N (V_{Gr}=0.04%) the extrapolated values deducted from the experimental $\mathrm{SE_T}$ curves as a function of frequency.

b) Decomposition of shielding effectiveness

The shielding effectiveness $\mathrm{SE_{TOT}} = -20log\mathrm{T}(\omega)$ is defined as the total attenuation (measured in dB) and consists of three contributions due to signal surface reflection, multiple internal reflections, and absorption. Under the good conductor approximation ($\sigma \gg \omega\epsilon$, where ω is the angular frequency and ϵ the material dielectric constant), $\mathrm{SE_{TOT}}$ can be estimated using a simplified expression:

$$\mathrm{SE_{TOT}} = 20\log_{10}\left(\frac{Z_0}{4}\sqrt{\frac{\sigma}{\omega\mu}}\right) + 20\log_{10}\left|1 - 10^{-\frac{\mathrm{SE_A}}{10}}\right| + \mathrm{SE_A} \qquad \text{Supplementary Equation (5)}$$

The first two terms refer to the contribution produced by the interface (air-sample, with Z_0 free space impedance) reflection and internal reflections respectively, whereas

$$\mathrm{SE_A} = 6.14\ \mathrm{t}\sqrt{\sigma\ \omega\ \mu} \qquad \text{Supplementary Equation (6)}$$

takes into account absorption losses inside the sample.

The first and third term are directly correlated with sample properties (electrical conductivity σ, magnetic permeability μ, thickness t). Since we are considering a non-magnetic material, in our case $\mu = \mu_0$, the free space permeability. The second term, SE_M, expressing attenuation due to multiple internal reflections, in layered systems is actually a negative term that reduces the total shielding effectiveness. When SE_A is larger than 10 dB, usually this contribution can be neglected, which is always the case in our specimens but at the smallest layer numbers (i.e. at small V_{Gr}). Therefore, the SE behaviour as a function of frequency depends on the interplay between the contributions to the shielding given by material absorption and interface reflection only. Indeed, the SE_A term obviously increases with frequency since it inversely depends on the penetration depth, whereas the SE_R term shows a decreasing frequency behaviour according to the transmission line model of shielding [7]. For the majority of samples, this implies that the two contributions balance out and the overall shielding is almost constant with frequency. However, in the limit of a good conductor approximation the electric field is actually "shorted out" at the first interface, and the SE_R term relative weight in Supplementary Equation (5) diminishes [8]. This is clearly seen in Fig. 4c for specimens having the same graphene volume but different total thickness: when absorption becomes the dominant shielding mechanism, SE as a consequence increases with frequency. Moreover, as expected from Supplementary Equation (6), the higher the conductivity the larger is the contribution given by SE_A. Nevertheless, a further increase in overall conductivity produced by reducing thickness of the single graphene/PMMA layer will produce a minor effect on the total shielding effectiveness since this term linearly depends on t. A high conductivity increases also the shielding SE_R produced by sample surface, however reflection and absorption follow an opposite trend as a function of frequency, therefore they tend to intersect at the highest frequencies.

In Supplementary Figure 7 we plot the total shielding effectiveness for the 100 layers sample (0.5 vol%) and its decomposition in the three different contributions SE_R, SE_M and SE_A in the frequency interval of investigation. It is worth to highlight that in all measurements presented here the frequency behaviour of the shielding effectiveness is consistent with a simple Drude scenario where the graphene electrical conductivity still shows a flat response in the low THz region, consistently with many literature reports [16-18]. Of course, it is possible that a frequency drop in the in-plane electrical conductivity and therefore in the SE dependence occurs at higher frequencies, in agreement with the predictions given in [6]. Moreover, as discussed in the main text, at the highest volume fraction there might be an additional contribution to the shielding given the presence of hopping electrons (through-plane conduction) when t_{PMMA} is less than 100 nm.

Supplementary Methods

Scratch test method

The thickness of the single PMMA/ CVD graphene layer was measured through atomic force microscopy (AFM) with a Dimension Icon (Bruker) instrument. The single PMMA/ CVD graphene layer deposited on Si wafer was scratched using a scalpel without damaging the substrate. AFM images of the scratch were acquired in the Peak Force Tapping mode using ScanAsyst-Air probes (stiffness 0.2−0.8 N/m, frequency ~80 kHz). Several scratches were measured for each deposited layer to allow statistical analysis of data. The average depth of the scratch below the mean surface plane, corresponding to the film thickness, was evaluated using the cross-section analysis of the Nanoscope Analysis software.

Scanning Electron Microscopy

Scanning Electron Microscopy (ZEISS SUPRA 35VP SEM) was employed to assess the morphology of the produced nanolaminates.

Supplementary Figures

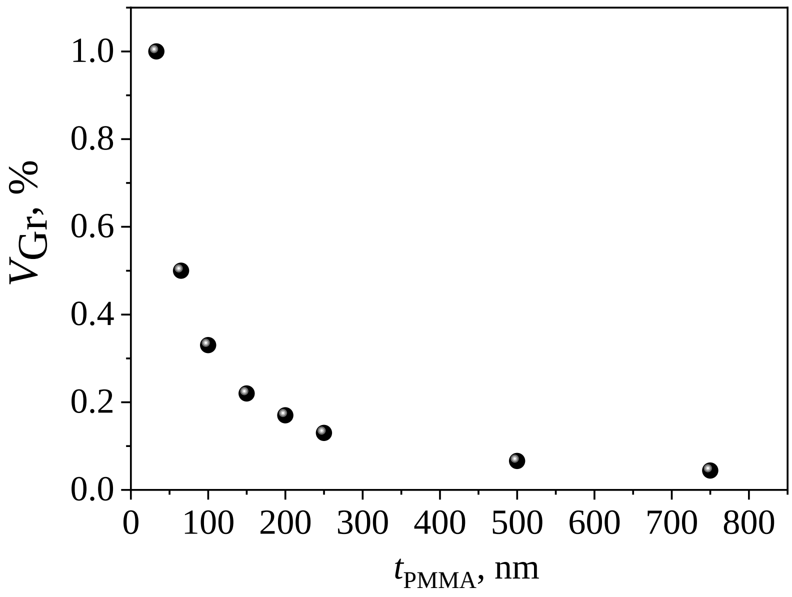

Supplementary Figure 1. Relation between the thickness of the polymeric layer and the volume fraction of graphene in the nanolaminate.

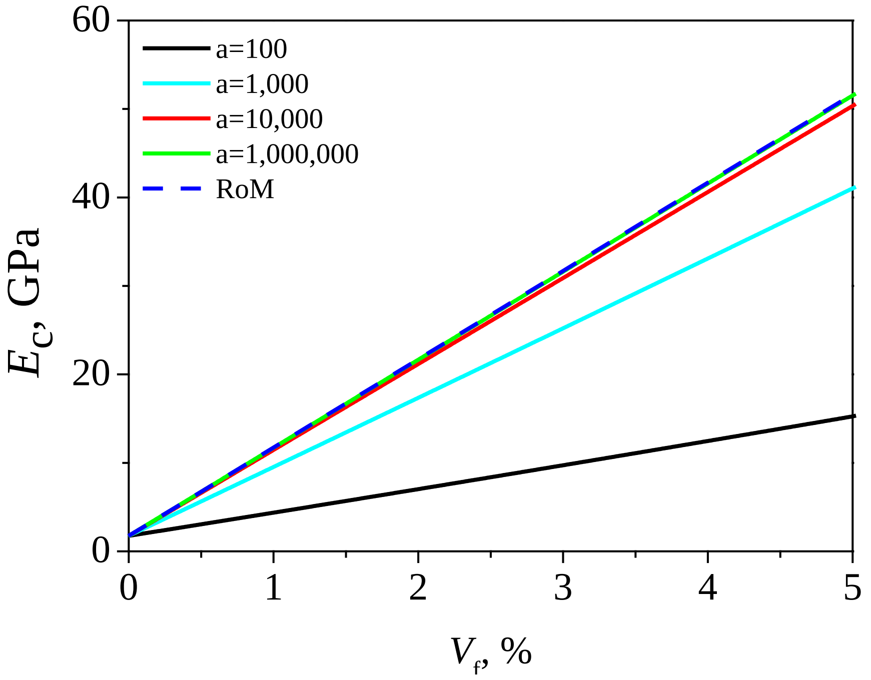

Supplementary Figure 2. Theoretical predictions of the elastic modulus of a graphene/PMMA nanocomposite (E_c) versus graphene volume fraction V_{Gr}: Halpin-Tsai model (solid lines) plotted for different graphene aspect ratios a and Rule of Mixture (dashed line).

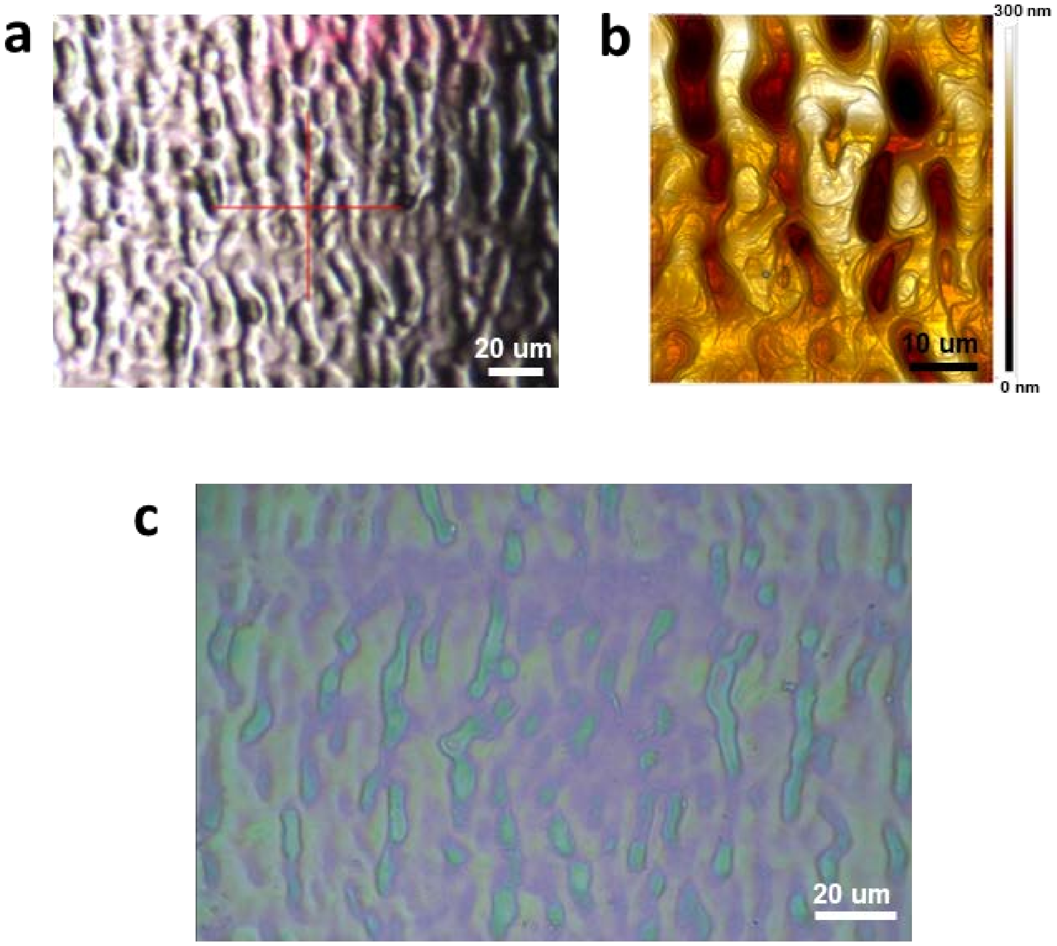

Supplementary Figure 3. Effect of roughness of copper foil on the morphology of the Gr/PMMA layer. (a) Optical micrography and (b) AFM images of copper foil after CVD process. (c) Optical micrography of Gr/PMMA single layer deposited on Si wafer.

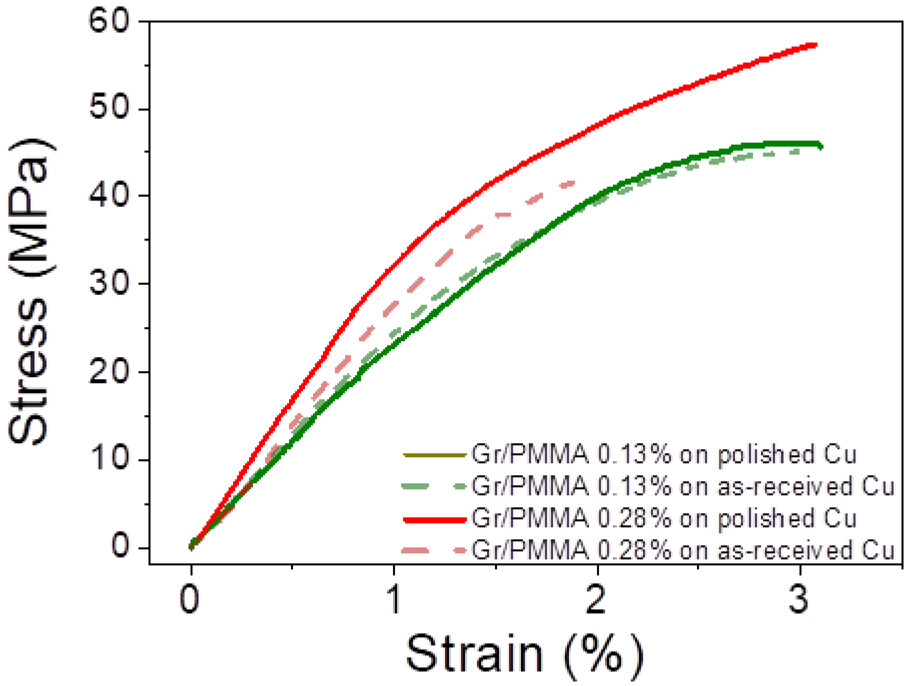

Supplementary Figure 4. Effect of roughness of Cu foil on the mechanical behaviour of the nanolaminates: Stress-strain curve of Gr/PMMA laminates produced on copper foils of different roughness. It is interesting noting that roughness of the Cu sacrificial substrate plays an increasing role as the thickness of the polymeric layer decreases. In fact, for the nanolaminates with 0.28% graphene (thickness of PMMA around 120 nm), a visible improvement of the mechanical performance is observed, while in the nanolaminates with 0.13 % (thickness of PMMA 250 nm), the effect of copper roughness is negligible on the final performance of the nanolaminate.

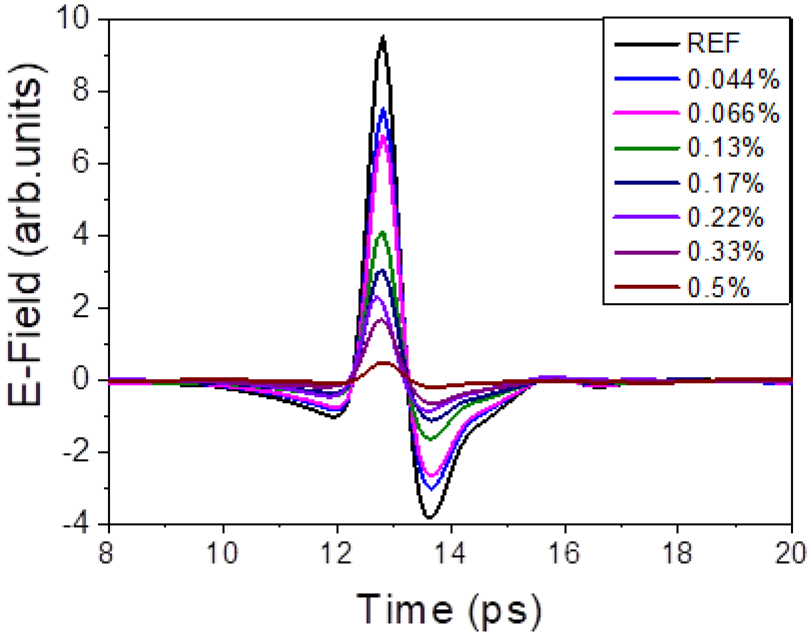

Supplementary Figure 5. THz transmission spectra for Gr/PMMA nanolaminates with different graphene content.

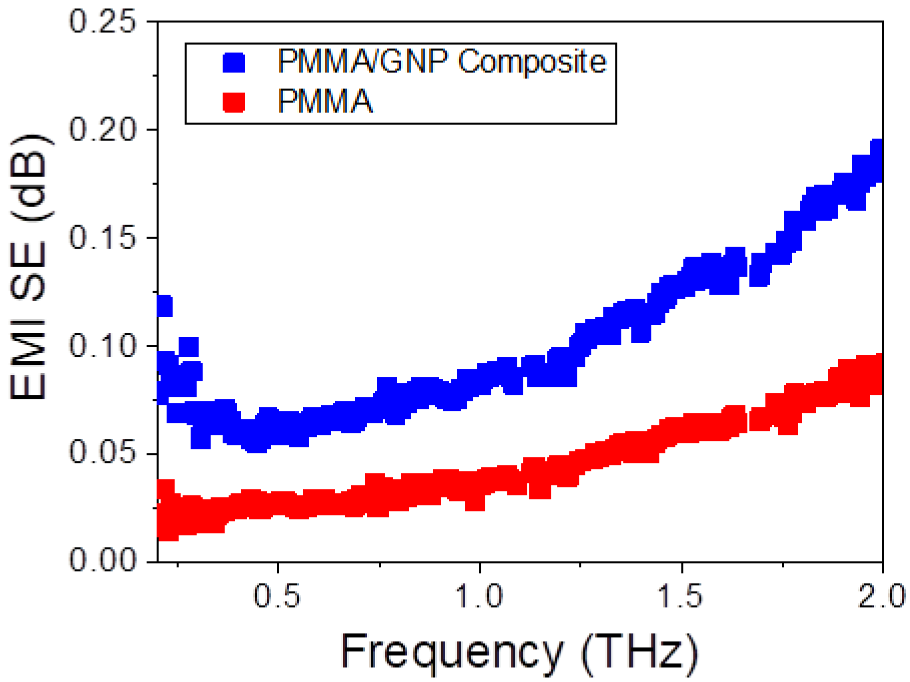

Supplementary Figure 6. Shielding effectiveness in the experimental frequency range for neat PMMA and PMMA filled with 0.27 vol% GNPs. The thickness of the films is 5 μm.

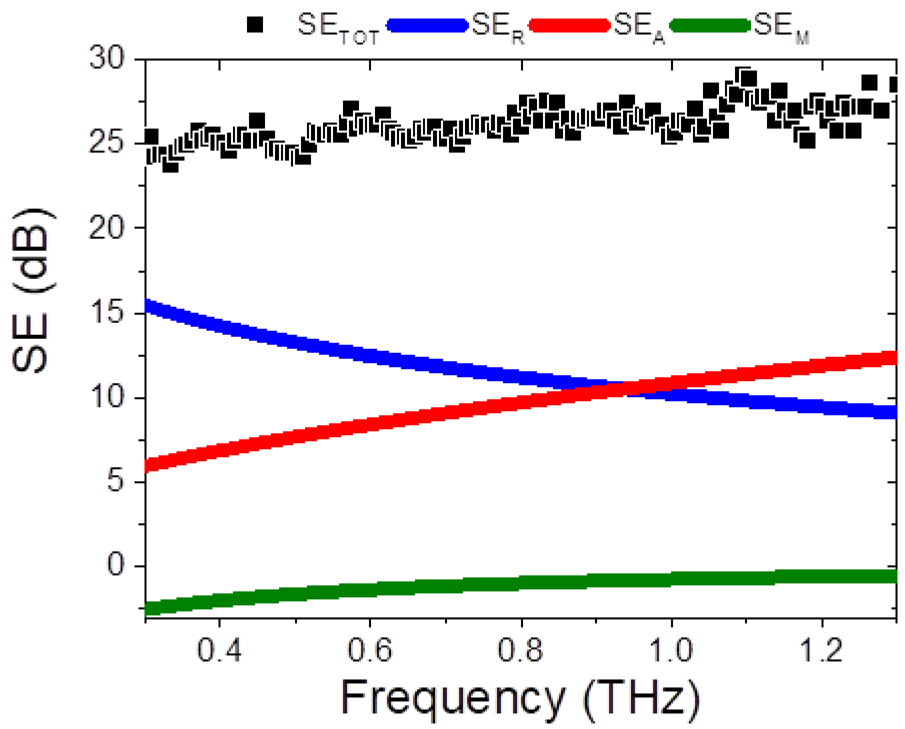

Supplementary Figure 7. Frequency dependence of the total (measured) shielding effectiveness SE_{TOT} (square symbols) and its decomposition in surface reflection, internal reflections and absorption contributions for 100 layers of graphene/PMMA stack with 0.5 vol% of graphene. SE_R, SE_A and SE_M (blue, red, and green continuous lines respectively) were calculated using Supplementary Eqs. (5) and (6).

Supplementary Tables

Supplementary Table 1. Fabrication details of Gr/PMMA nanolaminates

Solution in Anisole (wt%)	Angular speed (RPM)	Layer Thickness (nm)	Nominal Graphene Volume Fraction (%)	N. of layers	Nominal Nanolaminate thickness (µm)	Measured Final thickness (µm)	Actual Graphene Volume Fraction (%)
**3**	1500	250	0	20	5	5.05	0
**6**	1000	750	0.044	6	4.5	4.64	0.04
**6**	2000	500	0.066	10	5	5.15	0.06
**3**	1500	250	0.13	20	5	4.98	0.13
**3**	2000	200	0.165	20	4	4.1	0.17
**3**	3000	150	0.22	30	4.5	4.58	0.22
**2**	1500	100	0.33	50	5	5.08	0.33
**2**	3000	65	0.5	100	6.5	6.47	0.5
**1**	1500	33	1	4	0.132	0.132	1

Supplementary Table 2. State of the art on graphene-PMMA discontinuous composites

Filler	Vol. fraction (%)	E (GPa)	Tensile strength (MPa)	Elongation at break (%)	Conductivity (S/cm)	Ref.
rGO (in situ polymerization)	0	0.75	24.2	2.8	3.00E-11	9
	0.54	0.865	23	2.6	9.00E-06	
	1	0.85	14.2	1.5	8.00E-05	
rGO (sheet casting)	0.05				6.00E-07	
	0.27				8.00E-05	
	0.54	0.963	28.4	2.2	8.00E-04	
	1	1.054	26.6	1.7	1.00E-03	
CRGO	0	3.12	55.4	2.5	N.A.	10
	0.54	3.85	50.7	2.27		
PMMA-grafted-CRGO	0.54	4.43	63.8	2.46		
Graphene functionalized PMMA (in situ polymerization)	0	2.09	30.78	1.67	N.A.	11
	0.27	2.71	43.3	2.42		
	0.11	2.89	58.52	2.77		
	0.27	5.24	66.08	3.32		
	0.54	4.67	49.15	1.79		
PMMA grafted GO (solvent casting)	0	1.87	35.5	2.3	N.A.	12
	0.27	2.09	37.1	3.5		
	0.54	2.18	42	4.8		
	1.62	1.56	21.4	0.9		
non modified GO (solvent casting)	0.54	1.45	32.7	2.7		
FLG (5µm) (melt mixing)	0	2.35	59.5	4.15	N.A.	13
	0.29	2.43	60	4.1		
	0.58	2.47	60	4.1		
	1.22	2.55	61	3.9		
	3	2.6	61	3.8		
	5.9	2.5	59	3.1		
	11.5	2.8	55	3		
FLG (20 µm) (melt mixing)	1.22	2.62	60.5	3.8		
	3	2.85	60.5	2.1		
	5.9	3	59	3		
	11.5	4.1	42	1.6		
GO (melt mixing)	0	2.55	61	3.9	N.A.	14
	0.29	2.75	60	3		
	0.58	3	59	3.1		
	1.22	2.9	60	3.2		
	3	2.6	59	2.8		
	5.9	2.85	31	1.5		
Thermally exfoliated GO (solvent casting)	0	N.A.	N.A.	N.A.	7.5E-17	15
	0.05				8.5E-17	
	0.26				3.33E-16	
	0.52				3.33E-14	
	0.78				2.38E-4	
	1.05				5E-3	
	1.58				1E-3	
	2.67				1E-2	

Supplementary Table 3. Comparison between minimum (theoretically calculated) and measured EMI Shielding Effectiveness.

**V_{Gr} [%]**	**σ [S/cm]**	**Nanolaminate thickness (μm)**	**Minimum shielding [dB]**	**SE@0.5 THZ [dB]**
0.04	4.6	4.64	3.1	2.9
0.06	6.9	5.15	4.0	7.4
0.13	8.7	4.98	5.2	10.1
0.22	16.7	4.58	8.2	12.4
0.33	28.7	5.08	11.4	15.3
0.5	93.1	6.47	21.9	23.2

Supplementary References

1. Voigt W, Uber die Beziehung zwischen den beiden Elastizitatskonstanten Isotroper Korper, Wied. Ann 38, 573-587 (1889).

2. Halpin JC, Kardos JL, Halpin–Tsai equations – review, Polym Eng Sci, 16, 344–52 (1976).

3. Lee C, Wei X, Kysar JW, & Hone J, Measurement of the elastic properties and intrinsic strength of monolayer graphene, Science, 321(5887), 385-388 (2008).

4. Liu P, Jin Z, Katsukis G, et al., Layered and scrolled nanocomposites with aligned semi-infinite graphene inclusions at the platelet limit, Science, 353(6297), 364-367 (2016).

5. Saini P and Arora M, Microwave absorption and EMI shielding of nanocomposites based on intrinsically conducting polymers, graphene and carbon nanotubes, in New Polymers for Special Applications, Chap. 3, IntechOpen (2012).

6. D'Aloia AG, D'Amore M, and Sarto MS, Optimal terahertz shielding performances of flexible multilayer screens based on chemically doped graphene on polymer substrate, IEEE International Symposium on Electromagnetic Compatibility, 2015-September, art. no. 7256309, 1030-1035 (2015).

7. Schelkunoff SA, The impedance concept and its application to problems of reflection, refraction, shielding and power absorption, Bell. Syst. Tech. J. 17, 1, 17-48 (1938).

8. Paul CR, “Introduction to Electromagnetic Compatibility”, Second Edition, Wiley & Sons, Inc. (2006).

9. Tripathi SN, Saini P, Gupta D, Choudhary V, Electrical and mechanical properties of PMMA/reduced graphene oxide nanocomposites prepared via in situ polymerization, J. Mater. Sci. 48, 6223-6232 (2013).

10. Gong LX, Pei Y-B, Han Q-Y, Zhao L, Wu L-B, Jiang J-X, Tang L-C, Polymer grafted reduced graphene oxide sheets for improving stress transfer in polymer composites, Comp. Sci. Technol. 134, 144-152 (2016).

11. Wang J, Shi Z, Ge Y, Wang Y, Fan J, Yin J, Solvent exfoliated graphene for reinforcement of PMMA composites prepared by in situ polymerization, Mater. Chem. Phys. 136, 43-50 (2012).

12. Gonçalves G, Marques PAAP, Barros-Timmons A, Bdkin I, Singh MK, Emamic N and Grácioa J, Graphene oxide modified with PMMA via ATRP as a reinforcement filler, J. Mater. Chem. 20, 9927-9934 (2010).

13. Vallés C, Abdelkader AM, Young RJ, Kinloch IA, The effect of flake diameter on the reinforcement of few-layer graphene–PMMA composites, Comp. Sci. Technol. 111, 17-22 (2015).

14. Vallés C, Kinloch IA, Young RJ, Wilson NR, Rourke JP, Graphene oxide and base-washed graphene oxide as reinforcements in PMMA nanocomposites, Comp. Sci. Technol. 88, 158-164 (2013).

15. Zhang H-B, Zheng W-G, Yan Q, Jiang Z-G, Yu Z-Z, The effect of surface chemistry of graphene on rheological and electrical properties of polymethylmethacrylate composites, Carbon 50, 5117-5125 (2012).

16. Tomaino JL, Jameson AD, Kevek JW, Paul MJ, van der Zande AM, Barton RA, McEuen PL, Minot ED, and Lee Y-S, Terahertz imaging and spectroscopy of large- area single-layer graphene, Opt. Express 19, 141-146 (2011).

17. Maeng I, Lim S, Chae SJ, Lee YH, Choi H, and Son J-H, Gate-Controlled Nonlinear Conductivity of Dirac Fermion in Graphene Field-Effect Transistors Measured by Terahertz Time- Domain Spectroscopy, Nano Lett. 12, 551-555 (2012).

18. Whelan PR, Huang D, Mackenzie D, Messina SA, Li Z, Li X, Li Y, Booth TJ, Jepsen PU, Shi H, and Bøggild P, Conductivity mapping of graphene on polymeric films by terahertz time-domain spectroscopy, Opt. Express 26, 17748-17754 (2018).
